# Antiviral factors and type I/III interferon expression associated with regulatory factors in the oral epithelial cells from HIV-1-serodiscordant couples

**DOI:** 10.1038/srep25875

**Published:** 2016-05-11

**Authors:** Cesar A. C. Cervantes, Luanda M. S. Oliveira, Kelly C. G. Manfrere, Josenilson F. Lima, Natalli Z. Pereira, Alberto J. S. Duarte, Maria N. Sato

**Affiliations:** 1 Laboratory of Dermatology and Immunodeficiencies, LIM-56, Department of Dermatology, Institute of Tropical Medicine, University of São Paulo, São Paulo, Brazil

## Abstract

Individuals who remain HIV-seronegative despite repeated unprotected exposure to the virus are defined as exposed seronegative (ESN) individuals. Innate and adaptive immunity, as well as genetic factors, provide ESNs with important advantages that allow for low infection susceptibility. The majority of HIV-1-infected individuals undergo antiretroviral therapy, which can decrease the level of HIV-1 exposure in ESNs. We analyzed type I interferon (IFN)-related antiviral and regulatory factors in peripheral blood mononuclear cells (PBMCs) and oral epithelial cells from serodiscordant couples. Our findings revealed that ESNs did not induce the expression of antiviral factors (APOBEC-3G, TRIM5-α, SAMDH1, STING, TBk1) or regulatory factors (Trex, Foxo3, Socs3, IL-10) in PBMCs, unlike their HIV-1-infected partners. In contrast, ESNs upregulated APOBEC-3G and type I/III IFNs (IFNs-α,-β/-λ) in oral mucosal epithelial cells similar to their HIV-infected partners. The serodiscordant groups exhibited an increased expression of type I IFN-induced regulators, such as Trex and Foxo3, in oral epithelial cells. TLR7, TLR8 and TLR9 were expressed in oral epithelial cells of both ESNs and HIV-1-infected subjects. These findings revealed evidence of antiviral factors, type I/III interferon and regulatory factor expression only in the oral mucosal compartment of ESNs, while HIV-1-infected partners systemically and oral mucosal expressed the antiviral profile.

High-risk populations (e.g., sex workers, serodiscordant couples, intravenous drug users, and children born from HIV-1-infected mothers) have been studied as exposed seronegative (ESN) individuals^1^,^2^,^3^,^4^.

The mechanisms involved in the protection against HIV infection are multifactorial, and the combined contributions of innate and adaptive immunity, as well as genetic factors, provide important advantages that allow for a low susceptibility to infection^5^,^6^,^7^. Mucosal and systemic HIV-1-specific cellular and humoral responses may play defining roles in the protection against HIV-1 infection^8^,^9^,^10^,^11^,^12^. By analyzing the innate immunity of ESNs, we verified that natural killer (NK) cells presented a unique activation profile, with increased levels of NKG2D, CD107a, and interferon (IFN)-γ expression and memory CD57^+^CD56^dim^ NK cells^13^. We also found that ESNs contained populations of Tc22/Th22 cells and polyfunctional staphylococcal enterotoxin B-induced CD4^+^ and CD8^+^ T cells with a low activation profile^14^. Moreover, a dysfunctional innate immune response to Toll-like receptor (TLR) stimulation has been detected in HIV-1-infected mothers treated with antiretroviral therapy (ART) and their newborns^15^. We also observed a significant increase in mRNA expression of antiviral factors in mononuclear cells from HIV-infected mothers and cord blood compared to uninfected mother-newborn pairs, including the apolipoprotein B mRNA-editing enzyme 3G (A3G), A3F, tripartite motif family-5α (TRIM-5α), TRIM-22, myxovirus resistance protein A (MxA), stimulator of IFN genes (STING) and IFN-β^16^.

HIV replication is limited by cellular restriction factors, such as the citidine deaminase A3G, which acts by inducing G to A mutations^17^. ESNs expressed higher levels of A3G than healthy controls, suggesting that exposure to HIV may trigger A3G expression in peripheral blood mononuclear cells (PBMCs) in the absence of infection^18^. The SAMHD1 (SAM domain and HD domain-containing protein 1) is a deoxynucleoside triphosphate triphosphohydrolase that degrades the intracellular pool of deoxynucleoside triphosphates available during early reverse transcription^19^. Moreover, TRIM-5α may block infection at the post-entry pre-integration phase by promoting viral capsid degradation^20^.

Type I and II IFN induce the expression of many antiviral proteins. STING is a critical signaling molecule involved in the innate response to cytosolic nucleic acid ligands and is also thought to sense membrane fusion events associated with viral entry independent of nucleic acid sensing^21^. TANK-binding kinase 1 (TBk1) is a key serine/threonine protein kinase in the IFN signaling pathway, and HIV-1 can block IFN induction by inhibiting TBk1 function^22^. In addition to the type I IFN-mediated antiviral response, the induction of regulatory factors is crucial in controlling the inflammatory response. Notable factors include Trex, a regulator of lysosomal biogenesis and IFN-independent activation of antiviral genes^23^, Foxo3, a member of the forkhead family of transcription factors and a negative regulator of IRF7 transcription^24^, suppressor of cytokine signaling 3 (Socs 3)^25^, and IL-10, which inhibits antiviral IFN-β signaling. The balance between antiviral type I IFN and the expression of IFN-regulatory factors in ESNs is interesting to evaluate considering their low CD4^+^ T-cell activation profile.

Mucosal epithelial cells, such as those found in the oral cavity, serve as an important compartment in sexual intercourse. The risk of HIV transmission during oral sex is very low^26^, and certain antimicrobial peptides, such as human beta defensins, have been implicated in the low rate of oral HIV transmission^27^. Orally exposed uninfected men who have had sex with men can induce neutralizing anti-HIV-1 activity in plasma, which is primarily mediated by non-HIV-envelope-specific IgA1^28^. HIV exposure through oral sex is sufficient to induce systemic HIV-specific CD4^+^ and CD8^+^ T-cell immune responses in some uninfected individuals^29^. High mRNA levels of antiviral factors such as ELAFIN, SAMHD1, SerpinA1 and A3G have been found in the oral mucosa of ESNs^30^. Moreover, due to the constant presence of commensal microbes in the oral cavity, TLR expression may have an important function in oral tissue homeostasis^31^.

Evaluation of the PBMCs and oral epithelial cells of serodiscordant couples with low HIV-1 exposure for the transcriptional expression of factors involved in natural resistance to HIV infection, including antiviral factors, such as type I and III IFN, regulatory factors, and antiviral response-related TLRs, may be a novel approach to identify vaccine adjuvants.

## Results

### Antiviral and regulatory factor expression in PBMCs and oral epithelial cells of serodiscordant couples

The serodiscordant couples had long-term relationships of approximately 13 years, and the average time since initial HIV-1 diagnosis was 9.5 years (Table 1). At the start of the study, ESNs exhibited negative serology for HIV-1. The majority (15/16) of HIV-1-infected partners were receiving ART treatment. Of these subjects, only 4 had detectable VLs, demonstrating a persistent low exposure to HIV-1. Moreover, the majority of couples in our cohort were heterosexual (68.7%, 11/16 couples).

ESNs individuals have been previously shown to express higher levels of A3G in peripheral blood cells than healthy controls (HCs), and A3G expression in ESNs significantly decreased a year after HIV diagnosis and subsequent treatment of their partners^18^. There is a growing population of stable ESN individuals who have chronically ART-treated partners, and the profile of antiviral and regulatory factor expression in this group is unknown. We performed qPCR analysis of antiviral restriction factors (A3G, TRIM-5α, SAMDH1), IFN type I pathway-related factors (STING, TBk1 and IFN-β) and regulatory factors (Trex, Foxo3, Socs3, IL-10) in the PBMCs of ESNs and their HIV-1-infected partners, as well as in HC individuals.

**Figure 1:**
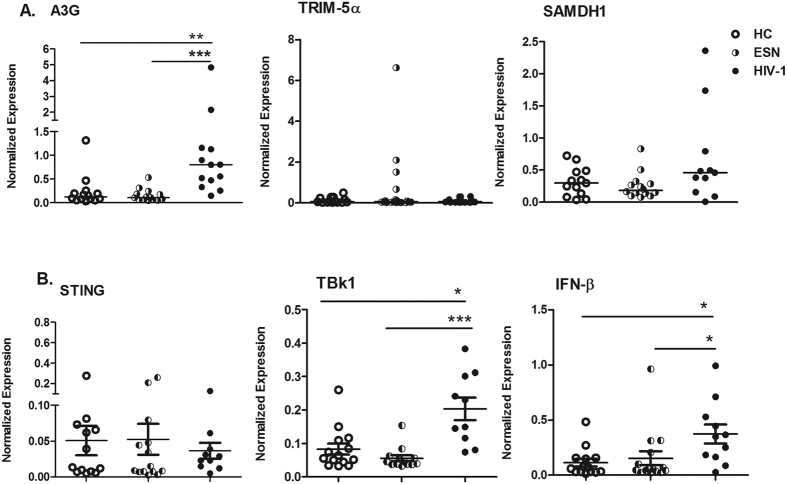
Increased antiviral restriction factor expression in PBMCs of HIV-1-infected partners from serodiscordant couples. Real-time PCR analysis of mRNA expression levels of antiviral factors, such as (**A**) A3G, TRIM-5α, SAMDH1, (**B**) STING, TBk1, and IFN-β in PBMCs of healthy controls (HCs, n = 15), exposed seronegative (ESNs, n = 14) individuals and the corresponding HIV-1 infected partners (n = 13). The data represent the median values. *p ≤ 0.05, **p ≤ 0.01, ***p ≤ 0.001.

**Figure 2:**
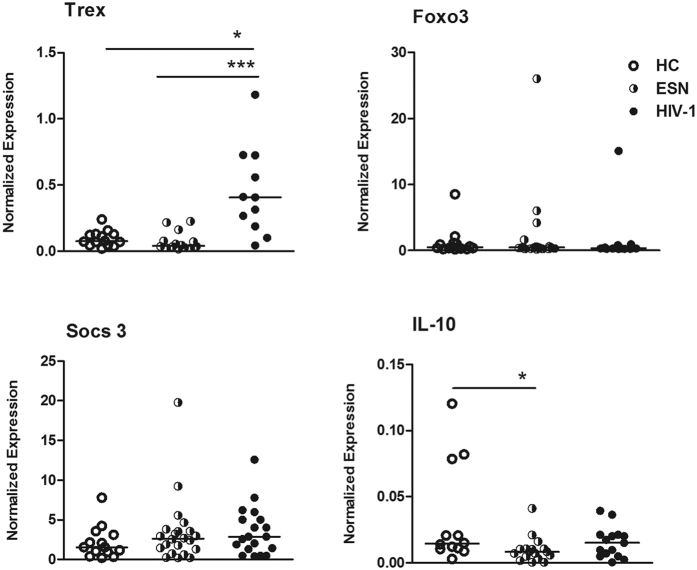
Expression of regulatory factors in PBMCs of serodiscordant couples. The mRNA expression levels of Trex, Foxo3, Socs3 and IL-10 in PBMCs of healthy controls (HCs, n = 14), exposed seronegative (ESNs, n = 11–15) individuals and the corresponding HIV-1 infected partners (n = 11–15) were evaluated by real-time PCR. The data represent the median values. *p ≤ 0.05, ***p ≤ 0.001.

Figure 1A indicates that A3G expression increased in PBMCs from HIV-1-infected individuals compared to the HC and ESN individuals. A subset of ESNs (4/16) had detectable TRIM-5α expression, although there were no significant differences between the groups. Moreover, TBk1 and IFN-β expression increased in HIV-1-infected individuals (Fig. 1B). We next evaluated factors linked to the regulation of IFN-stimulated gene (ISG) transcription and the duration of the type I response, such as Trex, Foxo3, Socs3 and IL-10, in the PBMCs of serodiscordant couples. Figure 2 illustrates that Trex expression was higher in HIV-1-infected partners than in HC and ESN individuals, whereas IL-10 expression was lower in the ESN group than in the HC group.

To determine whether the expression of these factors varied according to VL, we examined a group of HIV-1-infected progressor individuals (up to 5,000 copies/μL, in which 2/5 were treated with ART), as the majority of the HIV-1 partners in the initial cohort had undetectable viremia. The individuals in the additional HIV-1 viremic group were not related to the serodiscordant couples. As shown in Supplementary Figure 1, TBk1 expression did not correlate with viremia because both HIV-1-infected partners and viremic individuals exhibited increased expression levels. In contrast, only STING expression was pronounced in the HIV-1 progressors among the analyzed factors, demonstrating that viral replication likely triggers expression of this factor.

These results suggest that ESNs with low HIV exposure did not trigger antiviral or regulatory factor expression in PBMCs.

### Profile of antiviral factor expression in oral epithelial cells of ESN individuals

Because the cohort of serodiscordant couples reported engaging in oral intercourse, we evaluated antiviral factor expression in oral epithelial cells. Although we did not detect changes in PBMCs from ESNs, antiviral factor expression was pronounced in oral epithelial cells.

**Figure 3:**
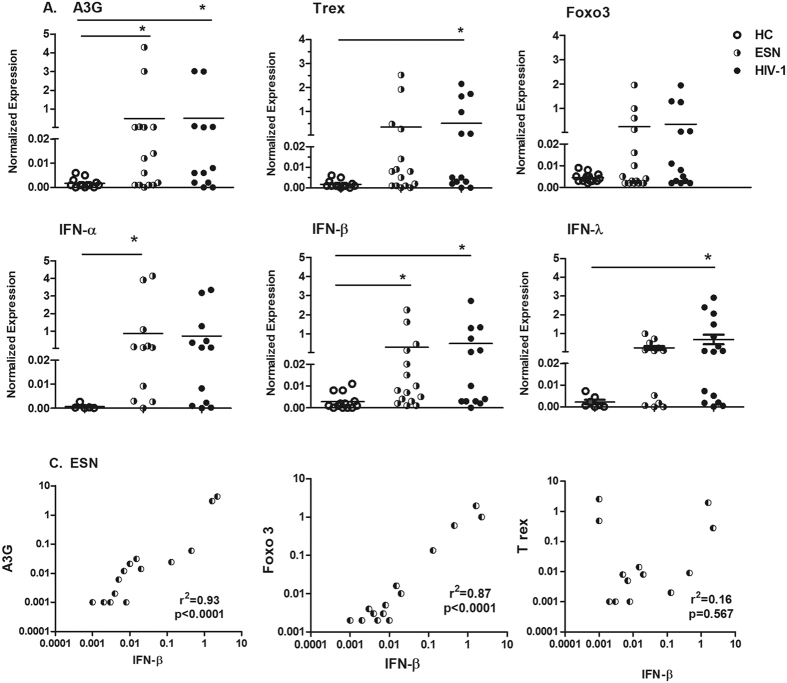
The upregulation of antiviral factor expression in the mucosal oral epithelial cells of serodiscordant couples. The mRNA expression levels of (**A**) A3G, Trex, Foxo3 and (**B**) IFN-α, IFN-β and IFN-λ are shown. (**C**) Correlations between ESN IFN-β expression and antiviral factors are presented. Cells from buccal washes were obtained from healthy controls (HCs, n = 10–14), exposed seronegative individuals (ESNs, n = 11–15) and HIV-1-infected partners (n = 11–15), and assessed by real-time PCR. The data represent the median values. *p ≤ 0.05.

Notably, we detected a similar mRNA expression profile of antiviral factors in both ESN and HIV-infected partners (Fig. 3). Serodiscordant couples exhibited increased A3G and IFN-β expression levels compared to HC individuals. IFN-α expression was increased in ESNs, and IFN-λ (type III IFN) was increased in HIV-1-infected individuals compared to HC individuals, although some serodiscordant samples were equally upregulated. The increased antiviral factor expression in the ESN group did not correlate with the VL status of their HIV-infected partner. We could not detect some antiviral factors in buccal epithelial cells, such as TRIM5-α, SAMDH1, STING and the regulatory factors Socs3, TBk1 and IL-10.

Moreover, we identified a positive correlation between A3G and Foxo3 expression with IFN-β in ESNs (Fig. 3C).

**Figure 4:**
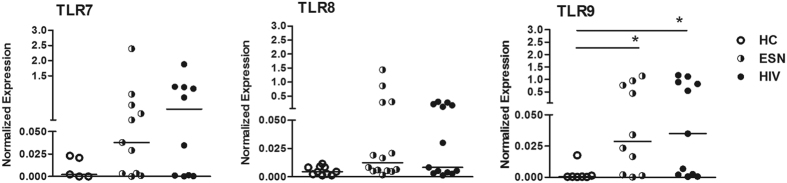
Increased TLR7, TLR8 and TLR9 expression in the mucosal oral epithelial cells from serodiscordant couples. The antiviral mRNA expression levels in cells from buccal washes from healthy controls (HCs, n = 5–10), exposed seronegative individuals (ESNs, n = 13) and HIV-1-infected partners (n = 13) were evaluated by real-time PCR. The data represent the median values. *p ≤ 0.05.

Because we identified a relevant antiviral expression pattern, we evaluated the expression of TLRs, such as TLR3, TLR7, TLR8, and TLR9, which are associated with the antiviral response. As shown in Fig. 4, TLR7, TLR8 and TLR9 were expressed at detectable levels in epithelial cells of serodiscordant couples, and TLR9 expression was increased compared to HC individuals. We did not detect TLR3 expression in oral epithelial cells.

These findings demonstrate the importance of evaluating mucosal compartments, such as the oral epithelium, in ESNs. Notably, the mucosal compartment triggered the expression of inducible factors related to type I and III IFNs, as well as type I IFN regulatory factors.

## Materials and methods

### Study subjects

HIV-1-serodiscordant couples from an outpatient clinic at the Emílio Ribas Infectious Diseases Institute in São Paulo, the Ambulatory Service of the Department of Secondary Immunodeficiency Clinic of the Clinical Hospital, University of São Paulo Medical School (HC/FMUSP) and the Centro de Referência e Tratamento em DST-AIDS in São Paulo, Brazil were enrolled into the following groups: ESN group (n = 16), HIV-infected partner (n = 16), or healthy donors (n = 14). Homosexual (n = 5) and heterosexual couples (n = 11) reported being with a single partner for more than 1 year. The couples reported participating in vaginal and oral sex at a frequency of 3–4 times per month, including unprotected sexual events. The required entry criteria included at least 1 high-risk sexual exposure per month in the past 12 months.

An additional group of HIV-1-infected individuals unrelated to the serodiscordant couples (n = 5, 1 female/4 males) with high viremic levels (>5,000 copies RNA/mL) were included, two of whom were on ART (mean, 57,224 copies RNA/mL).

All subjects provided written informed consent under the approval of the São Paulo University Institutional Use Committee (CAPPesq n°34871914.9.3001.5467). All methods used in this study were carried out in accordance with the approved guidelines, and all experimental protocols were approved by Hospital das Clínicas, School of Medicine, University of São Paulo. Standard eligibility criteria were used for enrollment into the study. Exclusion criteria included the use of immunosuppressant or immune-modifying drugs or pregnancy. The inclusion criteria included being over 18 years of age, reporting participation in unprotected sex and having a single partner for over 1 year. All the study subjects provided written informed consent under the approval of the São Paulo University Institutional Ethics Committee.

### Collection of buccal cells and PBMC

Buccal washes were performed by rinsing with 10 mL saline solution. The lavage fluid was centrifuged, and the supernatant was stored at −70 °C. The cellular pellet was stored in RNAlater (Sigma, St. Louis, MO, USA) at −20 °C. PBMCs were isolated from heparinized venous blood via Ficoll-Hypaque gradient centrifugation (GE Healthcare Bio-Sciences AB, Uppsala, Sweden) and stored in RNAlater at −20 °C.

### Real-time PCR

Total RNA was extracted from PBMCs and oral epithelial cells using an RNeasyPlus Mini Kit (Qiagen, Valencia, CA, USA), and reverse transcription was performed with a Sensiscript Reverse Transcriptase Kit (Qiagen). The primers used in the real-time PCR assay are detailed in Supplementary Table 1.

GAPDH and β-actin mRNA levels were used to normalize mRNA content from the PBMCs and oral epithelial cells, respectively. PCR was performed in an Applied Biosystems 7500 system using specific primers and SYBR Green fluorescence detection reagents (Applied Biosystems, Carlsbad, CA, USA). The cycling protocol consisted of 10 min at 95 °C, followed by 40 cycles of 15 s at 95 °C and 60 s at 60 °C. The amplification results were visualized and analyzed using Sequence Detection System (SDS) software (Applied Biosystems). Normalized expression was calculated as described previously by Livak^32^.

### Statistical analysis

Kruskal-Wallis tests with Dunn’s post-test were used to compare variables among HIV-infected individuals, ESNs and healthy controls. A p value ≤ 0.05 was considered statistically significant.

## Discussion

Our findings demonstrated that low HIV-1 exposure did not induce the expression of antiviral factors or regulatory factors in the PBMCs of ESNs, who exhibited an expression profile similar to uninfected controls. However, the oral mucosal epithelial cells from ESNs and their HIV-infected partners upregulated antiviral factors. The oral mucosal cells from serodiscordant groups had increased transcriptional expression of types I and III IFNs associated with type I IFN regulators, such as Trex and Foxo3. Moreover, the activation of innate immunity through increased TLR7, TLR8 and TLR-9 expression was detectable in oral epithelial cells from both ESNs and HIV-1-infected subjects. Serodiscordant couples exhibited similar expression profiles of antiviral, IFN type I/III and TLR receptors, which are all known to be regulated by type I interferon signaling. Although we observed a similar status of antiviral factors in oral cells between serodiscordant couples at systemic levels, only HIV-infected partners showed increased transcriptional expression in PBMCs. A high HIV exposure is likely required to trigger systemic antiviral factor expression, and the majority of the HIV-1 partners in this study had an undetectable viral load. These findings revealed that chronic low HIV-1 exposure could trigger the expression of innate immune response factors and regulatory factors in the oral mucosa.

The transcriptional expression of A3G, TBk1 and IFN-β, but not TRIM5α and SAMDH1, increased in mononuclear cells of HIV-infected partners compared to HCs and ESNs. In contrast, another model of newborn exposure to HIV-1 from HIV-1 infected mothers previously demonstrated the upregulation of A3G, TRIM-5α, SAMDH1, STING, and IFN-β in the PBMCs of mothers, in the cord blood cells and in the placental tissue compared to mother-newborn uninfected individuals^16^. This finding suggests that even when HIV-1 is suppressed in mothers by ART, there is a high probability of HIV exposure and the induction of antiviral factor expression in newborns, whereas the ESN cohort revealed low but chronic HIV exposure.

Decreased A3G expression in the PBMCs from ESNs has been associated with the cessation of exposure^18^. It is possible that the degree of exposure and the VL of the HIV-1-infected partner can influence antiviral levels. Indeed, A3G and TBk1 expression was induced regardless of the VL of the HIV-infected partners, whereas the induction of STING was dependent on viral replication, as STING expression was upregulated in the viremic individuals. STING is activated to recruit signaling cofactors such as TBk1 and IKK-α/β to subsequently activate IRF3 and NF-κB transcription factors and induce target gene expression, including HIV restriction factors, pro-IL-1β, type I IFN and proinflammatory cytokines and chemokines^33^.

We observed increased TBk1 and IFN-β expression in HIV-1-infected individuals. HIV-1 blocks type I and III IFN induction in myeloid cells through the two HIV-1 accessory proteins Vpr and Vif, which bind and inhibit TBk1, a key kinase in the IFN signaling pathway^22^. TBk1 regulates type I IFNs and NF-κB signal transduction^34^,^35^. TBk1 upregulation may prevent the inflammatory effects of type I IFN in HIV-infected subjects, making TBk1 upregulation a crucial regulatory mechanism because the long-term treatment of HIV has deleterious effects on immune activation^36^.

We identified a distinct transcriptional profile in the oral mucosal compartment compared to PBMC profile, in which A3G and types I (IFN-α and IFN-β) and III IFN (IFN λ) were upregulated in both ESNs and HIV-1-infected partners. In parallel, oral epithelial cells exhibited increased expression of regulatory factors such as Trex and Foxo3, suggesting an active role in regulating persistent inflammation and the immune activation role of IFNs, which may contribute to a low risk of HIV-1 infection in oral mucosal cells. The Foxo1/Foxo3 transcription factors regulate keratinocyte behavior and mediate the response to bacteria, such as *Porphyromonas gingivalis*^37^. In addition, the levels of chemokines CXCL10, CXCL9 and CCL2 were decreased in the saliva of ESNs compared to their HIV-infected partners, suggesting a low activation profile in ESNs (data not shown). Similarly, we previously verified that the polyfunctionality of CD4^+^ T cells in the ESN group was related to a less activated profile (CD38^−^) compared to the HIV-infected group (CD38^+^)^14^.

Type III interferons comprise 3 IFN-λ genes (IFN-λ1, IFN-λ2, and IFN-λ3) that encode 3 distinct but highly related proteins (IL-29, IL-28A and IL-28B). Type III interferons have antiviral activity against a broad spectrum of viruses and might contribute to the prevention of viral invasion through skin and mucosal surfaces^38^. IFN-λ3 exerts its anti-HIV function by activating JAK-STAT pathway-mediated innate immunity in macrophages^39^. In addition, IFN-λ has been shown to upregulate the intracellular expression of type I IFNs and A3G/3F^40^. IFN-α/β and IFN-λ bind distinct receptors but regulate similar sets of genes and exhibit strikingly similar biological activities. IFN-λ receptor expression is largely restricted to cells of epithelial origin. Because IFN-λ likely evolved to specifically protect epithelia, it is a relevant factor in the protection of the oral epithelial cells in ESNs and their HIV-infected partners.

We also observed increased TLR7, TLR8, and TLR9 levels in oral epithelial cells of ESNs and their HIV-infected partners, whereas we did not detect TLR3 expression. The upregulation of TLR9 in the ESN group may suggest that it plays an active role against other viruses in addition to HIV-1, such as double-stranded DNA viruses, including herpes viruses and cytomegalovirus (CMV). We previously found that ESNs demonstrated 100% seropositivity for CMV, similar to their HIV-infected partners, whereas the healthy group demonstrated 35% seronegativity. These findings correlated with the expansion of memory-like NK cells in ESNs^13^.

In clinical studies, the mixed TLR7/8 agonist resiquimod was topically applied to treat genital HSV or administered orally to treat HCV infection. However, the results revealed a lack of adequate efficacy and severe side effects at higher doses^41^. We reported that another TLR7/8 agonist, CL097, restored defective cytokine secretion by myeloid DCs from HIV-infected pregnant women and newborns^15^. Understanding the mechanisms involved in controlling TLR activation and regulation is crucial in establishing vaccine adjuvants for viral infections.

Oral keratinocytes on the mucosal surface are frequently exposed to HIV-1 through contact with infected sexual partners or nursing mothers. The oral mucosa is not as permissive for efficient HIV replication as other mucosal epithelia (e.g., vaginal/cervical and anal/rectal) and, therefore, may differ in susceptibility compared with these mucosal sites^42^. Although these cells do not express the common HIV-1 receptors and co-receptors found on permissive cells, it is unclear why oral keratinocytes support only nonproductive infection by HIV-1, while still harboring and transmitting infectious X4- or R5-tropic HIV-1 to permissive cells^43^. Uncovering the factors that explain the differential susceptibility and resistance to HIV infection in mucosal sites will allow for the identification and development of novel protective strategies.

The main factors triggered in HIV-1 and ESNs seem to be those regulated by IFN type I signaling, including antiviral factors and TLR7, 8, and 9 expression. These results showed that persistent low oral HIV-1 exposure may trigger antiviral factor expression and the regulatory factors that are able to avoid excessive inflammatory response. It is crucial for ESNs to avoid the inflammatory effects of IFNs to maintain the low T cell activation status^14^. Interestingly, the IFN-gene transcriptional profile between ESN and HIV-1 partners was similar in oral epithelial cells, though it remains to be determined whether they are distinct at the post-translational level.

Taken together, antiviral factors, such as types I and III IFNs and type I IFN regulatory factors, may be important to analyze in another compartment besides peripheral blood.

## Additional Information

**How to cite this article**: Cervantes, C. A. C. *et al*. Antiviral factors and type I/III interferon expression associated with regulatory factors in the oral epithelial cells from HIV-1-serodiscordant couples. *Sci. Rep.* **6**, 25875; doi: 10.1038/srep25875 (2016).

## Supplementary Material

Supplementary Information

## Figures and Tables

**Table 1:** Demographic characteristics of patients.

Groups	N	Gender	Years	CD4 (per mm^3^)	CD8 (per mm^3^)	Time Diagnostic Years	Time Relationship Years	Viral load (copies RNA/mL)
ESN	16	10F/6M	39 (33–45)	1268 (983–1496)	725 (498–1035)	–	13 (3–19,5)	–
HIV	16	1F/15M	40 (36–46)	633 (227–993)	674 (534–1126)	9.5 (2,2–14)	–	<50 (4/16)*
HC	14	10F/4M	39 (32–43)	1021 (782–1241)	606 (379–1020)	–	–	–

Results are shown as median and interquartiles. ESN: Exposed Seronegative, HIV: Infected HIV + , HC: Health Control. F: Female, M: Male. *Value represents detectable VL in 4/16 (51; 16,561; 17,511; 50,930 copies/mL).
