# Supplementary material for: Antiviral factors and type I/III interferon expression associated with regulatory factors in the oral epithelial cells from HIV-1-serodiscordant couples
Source: Sci Rep. 2016 May 11;6:25875. doi: 10.1038/srep25875 (PMC4863167; doi:10.1038/srep25875)
Supplement: Supplementary Information [file srep25875-s1.pdf]

Title: Antiviral factors and type I/III interferon expression associated with regulatory factors in the oral epithelial cells from HIV-1-serodiscordant couples

Authors: Cesar A.C. Cervantes, Luanda M. S. Oliveira, Kelly C.G. Manfrere, Josenilson F. Lima, Natalli Z. Pereira, Alberto J.S. Duarte, Maria N. Sato.

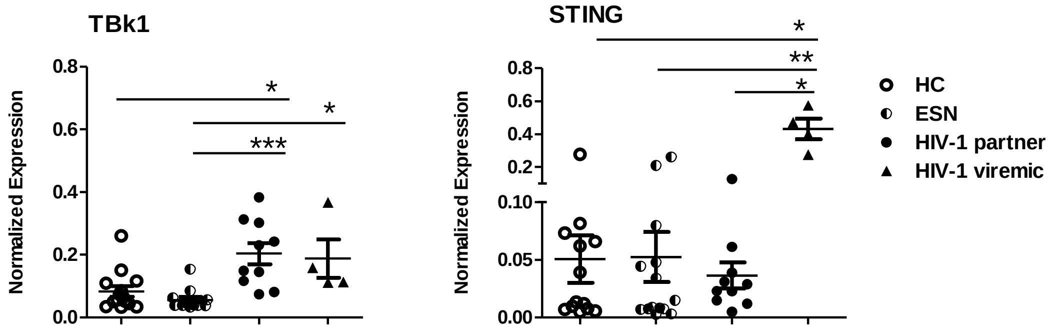

Supplementary Figure S1. Antivirals factors in the PBMCs of HIV-1 viremic subjects. The mRNA expression levels of TBk1 and STING in the PBMCs of healthy controls (HCs, n=14), HIV-1-exposed seronegative individuals (ESNs, n=13), HIV-1-infected partners (n=13), and viremic individuals (n=5) were evaluated by real-time PCR. The data represent median values. *p≤0.05, ** p≤0.01, ***p≤0.001.

**TLR-3**	Forward: 5’-GTG CCA GAA ACT TCC CAT GT-3’<br>Reverse: 5’-TCC AGC TGA ACC TGA GTT CC-3’
**TLR-7**	Forward: 5’-AAT GTC ACA GCC GTC CCT AC-3’<br>Reverse: 5’-GCG CAT CAA AAG CAT TTA CA-3’
**TLR-8**	Forward: 5’-TGT GAT GGT GGT GCT TCA AT-3’<br>Reverse: 5’-ATG CCC CAG AGG CTA TTT CT-3’
**TLR9**	Forward: 5’ - AAG GCC AGG TAA TTG TCA CGG - 3’<br>Reverse: 5’ – ACA ACA ACA TCC ACA GCC AAG T- 3’
**IFN-λ**	Forward: 5’-CGC CTT GGA AGA GTC ACT CA-3’<br>Reverse: 5’-GAA GCC TCA GGT CCC AAT TC-3’
**IFN-α**	Forward: 5’-AAA TAC AGC CCT TGT GCC TGG-3’<br>Reverse: 5’-GGT GAG CTG GCA TAC GAA TCA-3’
**IFN-β**	Forward: 5’-GGCTGGCCCTGTGATATTTCTGTG -3’<br>Reverse: 5’-ACCTGGCTCTCCTCCTCCCTTCCT -3’
**TRIM-5α**	Forward: 5’-CTG GCA TCC TGG GCT CTC AAA GT-3’<br>Reverse: 5’-CAT ACC CCC AGG ATC CAA GCA GTT-3’
**APOBEC3G**	Forward: 5’-GGCTCCACATAAACACGGTTTC-3’<br>Reverse: 5’-AAGGGAATCACGTCCAGGAA-3’
**SAMDH1**	Forward: 5’ - GGATTACTAAAAACCAGGTTTCACAACT – 3’<br>Reverse: 5’ - GCTCTGCAAATTTCTCTGGCAG - 3’
**STING**	Forward: 5’-ATATCTGCGGCTGATCCTGC -3’<br>Reverse: 5’-GGTCTGCTGGGGCAGTTTAT-3’
**TBK1**	Forward: 5´- GCAGTTTGTTTCTCTGTATGG - 3´<br>Reverse: 5´ - ATTGTTACCCCAATGCTCCA - 3’
**FOXO3**	Forward: TGG TGG CCT GAG ACA TCA AG<br>Reverse: ACG GTG TTC GGA CCT TAT C
**IL-10**	Forward: CAC ATG CGC CTT GAT GTC TG<br>Reverse: CAG GGC ACC CAG TCT GAG A
**TREX**	Forward: 5’ - GCCAAGACCTCTGCTGTCAC - 3´<br>Reverse: 5’ - CAGGGTCCTTCACTGGAGGAA - 3’
**SOCS3**	Forward: GCC CTG TCC AGC CCA ATA C<br>Reverse: AGG AAT CTA GCA GCG ATG GAA
**GAPDH**	Forward: 5′-GAA GGT GAA GGT CGG AGT-3′<br>Reverse: 5′-GAA GAT GGT GAT GGG ATT TC-3′
**β-actin**	Forward: 5'-CAT GTA CGT TGC TAT CCA GGC-3'<br>Reverse: 5’-CTC CTT AAT GTC ACG CAC GAT-3’

Supplementary Table S1- Oligonucleotide primers used in this study for PCR amplification.
